# Enhanced Antitumor Efficacy of Oncolytic Vaccinia Virus Therapy Through Keratin-Mediated Delivery in Triple-Negative Breast Cancer

**DOI:** 10.3390/ijms252111470

**Published:** 2024-10-25

**Authors:** Hyo-Sung Kim, Yun Hee Youn, Han-Jun Kim, Young-Hyun Koo, Junho Lee, Il Keun Kwon, Sun Hee Do

**Affiliations:** 1 Department of Veterinary Clinical Pathology, College of Veterinary Medicine, Konkuk University, 120 Neungdong-ro, Gwangjin-gu, Seoul 05029, Republic of Korea; 2 Department of Dental Materials, School of Dentistry, Kyung Hee University, 26 Kyungheedae-ro, Dongdaemun-gu, Seoul 02447, Republic of Korea; 3 College of Pharmacy, Korea University, 2511 Sejong-ro, Sejong 30019, Republic of Korea; 4 Department of Veterinary Surgery, College of Veterinary Medicine, Kyungpook National University, Daegu 41566, Republic of Korea; 5 Medical Device Research Center, Medical Science Research Institute, Kyung Hee University Medical Center, 23 Kyung Hee Dae-ro, Dongdaemun-gu, Seoul 02447, Republic of Korea

**Keywords:** triple-negative breast cancer, keratin, oncolytic vaccinia virus, delivery system

## Abstract

Triple-negative breast cancer (TNBC) represents an aggressive subtype characterized by high rates of recurrence and metastasis, necessitating the exploration of alternative treatment strategies. Oncolytic vaccinia virus (OVV) therapy has emerged as a promising approach, selectively infecting and destroying tumor cells. However, its efficacy is often hampered by inadequate viral distribution within the tumor microenvironment. Here, we investigate the potential of keratin (KTN) as a carrier for OVV delivery to enhance viral distribution and antitumor efficacy. In vitro assays revealed that KTN significantly improves OVV stability, leading to increased tumor cell apoptosis and necrosis. Furthermore, KTN effectively inhibits cancer cell migration by suppressing the epithelial–mesenchymal transition (EMT) process and downregulating metastasis-related proteins. These findings are corroborated in a syngeneic TNBC mouse model, where KTN-mediated OVV delivery enhances cytotoxic T cell-mediated antitumor immune responses without compromising the anti-angiogenic effects of the virus. Notably, KTN alone exhibits antitumor effects by suppressing tumor growth and metastasis, underscoring its potential as a standalone therapeutic agent. In conclusion, our study underscores the promise of KTN-mediated OVV delivery as a promising therapeutic strategy for TNBC. By improving viral distribution, suppressing EMT, and enhancing antitumor immunity, this approach holds significant potential for enhancing patient outcomes in TNBC treatment. Further investigation is warranted to explore the broader utility of KTN in various cancer therapy approaches.

## 1. Introduction

Triple-negative breast cancer (TNBC) represents a distinct subtype characterized by the absence of the estrogen receptor (ER), progesterone receptor (PR), and human epidermal growth factor receptor type 2 (HER2) [1,2]. Due to the lack of these targetable receptors, conventional treatment options for TNBC are limited primarily to chemotherapy [3]. However, TNBC is notorious for its high rates of recurrence, distant metastasis, and the associated adverse effects of prolonged chemotherapy, highlighting the urgent need for alternative therapeutic approaches.

Oncolytic viruses have garnered considerable attention as a promising strategy for treating various cancers, including bladder cancer, lung cancer, and pancreatic cancer [4,5,6,7]. Among these, the oncolytic vaccinia virus (OVV) stands out for its ability to selectively target, replicate within, and lyse tumor cells, which is facilitated by specific genetic modifications, such as the deletion of its thymidine kinase gene and vaccinia growth factor genes [8]. Additionally, the large size of the OVV (over 200 nm) enhances its tumor selectivity, as it faces challenges in extravasation from normal blood vessels but can penetrate the leaky vasculature that is characteristic of tumors [9].

In addition to its direct oncolytic activity, the OVV induces immunogenic cell death, releasing molecular patterns and cytokines that activate a cytotoxic immune response [10,11]. This triggers an inflammatory cascade capable of overcoming immune suppression within the tumor microenvironment [10]. Dendritic cells capture tumor-associated antigens from lysed cells, priming CD8+ T cells for a tumor-specific immune response [10]. Moreover, the direct viral invasion of endothelial cells, along with the recruitment of neutrophils and intravascular thrombosis mediated by cytokines and chemokines, may contribute to the destruction of tumor vasculature [12,13,14,15,16,17].

Despite its potential, the clinical efficacy of oncolytic viruses is often hampered by inefficient intratumoral spread and early viral clearance by the host immune systems [18,19]. To address these challenges, drug delivery systems have been developed to enhance intratumoral replication and improve therapeutic outcomes.

Keratin (KTN), a structural protein, not only provides mechanical stability but also facilitates intracellular signaling. Its high cysteine content allows for the formation of disulfide bonds, creating a stable matrix ideal for drug delivery [20,21]. Previous studies have demonstrated the potential of KTN derivatives in various applications, highlighting their accessibility, reproducibility, and biocompatibility, with reported antitumor efficacy in human melanoma cells and osteosarcoma [22]. Additionally, the role of a signal transducer and activator of transcription 3 (STAT3) in cancer biology is pivotal, as it regulates the genes involved in cell growth and apoptosis. By modulating STAT 3 activity, keratin-based delivery systems could potentially enhance the antitumor efficacy of the OVV through better immune modulation and direct tumor cell targeting.

In this study, we explore the development of a KTN-based delivery system for the OVV, aiming to enhance virus distribution and stability, thereby augmenting its antitumor effects. We characterize OVV–KTN solutions and evaluate the gene expression resulting from sustained OVV release. Antitumor efficacy is assessed using a 4T1 breast cancer mouse model, with a particular focus on immune infiltration and local viral replication, which is crucial for the success of antitumor therapy.

## 2. Results

### 2.1. KTN-Based Delivery System and Stability of OVV

**Figure 1:**
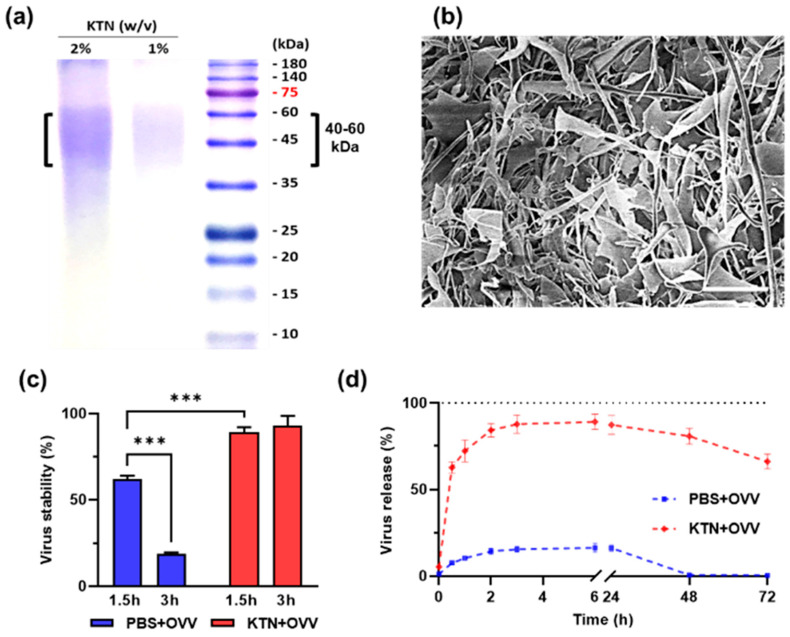
The keratin-based delivery system and stability of the oncolytic vaccinia virus. SDS-PAGE results for the two different concentrations of extracted KTN (**a**) and an SEM image of KTN powder after freeze-drying (**b**). The stability of the oncolytic vaccinia virus quantified via polymerase chain reaction (**c**). The quantification of the oncolytic vaccinia virus released through 3 μm pores from the keratin-OVV solution (**d**). Scale bars: 50 μm. The values are presented as the mean ± SD. A two-way ANOVA was followed by the Bonferroni post hoc test. *** *p* < 0.001.

To establish the KTN-based delivery system, the KTN was extracted from human hair using the Shindai method. Analysis of the extracted KTN was conducted using 2× sodium dodecyl sulfate–polyacrylamide gel electrophoresis (SDS-PAGE) and scanning electronic microscopy (SEM) to determine its molecular weight and morphology, respectively [23,24]. The molecular weight of the extracted KTN ranged between 40 and 60 kDa, which is characteristic of α-keratin (Figure 1a) [25,26]. SEM images revealed that the extracted KTN exhibited a scattered morphology composed of short protein fragments in the form of α-helical polypeptides (Figure 1b) [24,27].

The stability of the OVV in a KTN solution was assessed by incubating it at 37 °C in a CO_2_ incubator and analyzing virus particle counts using qPCR. Results demonstrated that the virus was more stable in a 1% keratin solution compared to Dulbecco’s phosphate-buffered saline (DPBS), and remained stable for 3 h (Figure 1c). Evaluation of the virus distribution in the KTN solution showed that the quantity of vaccinia virus particles passing through 3-μm pores reached its peak at 3 h in both PBS and KTN solutions (Figure 1d). However, after 3 days, 60% of the virus particles remained in the KTN solution, while none were detected in the PBS. These findings suggested that the OVV was more stable in the keratin solution and could be effectively distributed from the mixture.

### 2.2. In Vitro Anti-Proliferative Effect of OVV Combined with KTN

**Figure 2:**
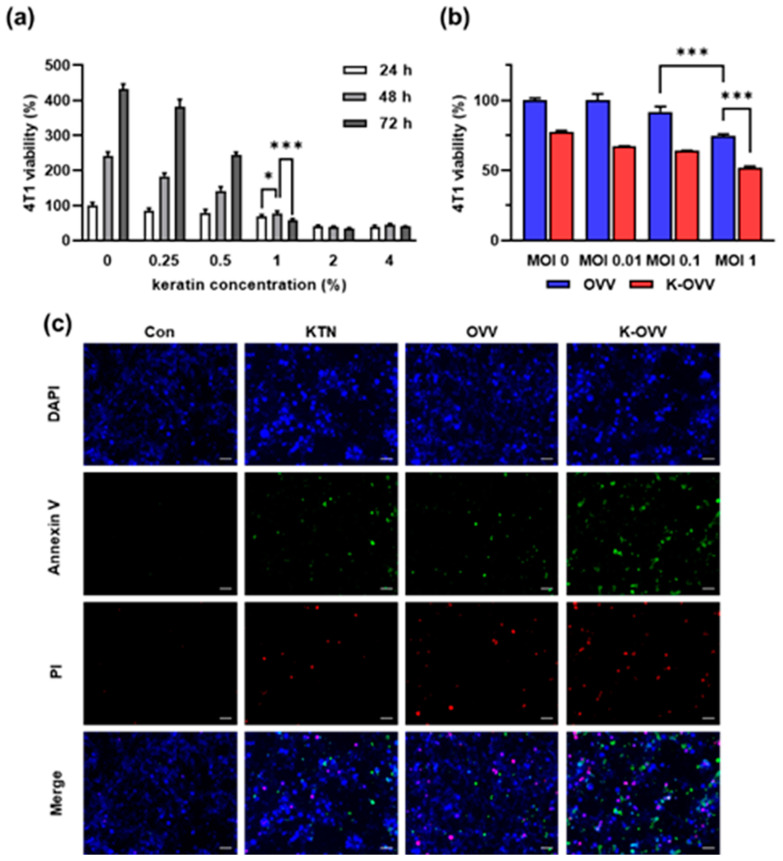
The in vitro antiproliferative effect of the OVV combined with the KTN. The viability of the 4T1 mouse breast cancer cells after treatment with various concentrations of the OVV in a keratin solution (**a**). The viability of the 4T1 cell line treated with different MOI of the OVV along with 1% keratin (**b**). The evaluation of apoptosis via Annexin V/PI staining in 4T1 cells treated with KTN and OVV at MOI 1 (**c**). Scale bars: 50 µm. The values are presented as the mean ± SD. A two-way ANOVA was followed by the Bonferroni post hoc test. * *p* < 0.05 and *** *p* < 0.001.

To determine the optimal concentration of KTN for OVV delivery, cell viability assays were performed using the 4T1 breast cancer cell line. Cell viability was determined via cell counting kit (CCK)-8 assays at 24, 48, and 72 h after treatment with DPBS or various concentrations (0%, 0.25%, 0.5%, 1%, 2%, and 4%) of KTN solution. Results indicated that 4T1 cells exhibited active proliferation at KTN concentrations below 0.5%, with the viability decreasing at higher KTN concentrations compared to the DPBS control group (Figure 2a). The 1% (*w*/*v*) KTN solution was selected for further analysis as it suppressed cell proliferation after 72 h.

The antitumor activity of the OVV in the KTN solution was evaluated by treating 4T1 cells with the OVV at a MOI of 0 to 1 loaded in DPBS or a 1% KTN solution. Cell viability decreased with higher OVV concentrations in both DPBS and the KTN solution (Figure 2b). Notably, cell viability in the OVV-only group (multiplicity of infection (MOI) = 1) and the KTN-only group (MOI = 0) was 75% and 78%, respectively. However, when cells treated with OVV were combined with KTN solution (K-OVV, MOI = 1), viability decreased to 52%, which is similar to the combined reductions seen in the OVV-only and KTN-only groups, suggesting an additive effect of KTN and OVV in reducing cancer cell viability.

Subsequently, Annexin V-FITC and PI staining were performed on 4T1 cells to assess apoptosis or necrosis (Figure 2c). The results showed positive staining for Annexin V or PI in cells treated with the KTN, OVV, or K-OVV. The K-OVV group exhibited higher levels of cell necrosis and apoptosis compared to the KTN or OVV group, which is consistent with the cell viability assay results. Moreover, staining revealed that only a few cells were stained with both Annexin V and PI, indicating that the KTN and the OVV induced both cell necrosis and apoptosis in the 4T1 cell line.

### 2.3. In Vitro Anti-Migratory Effect of OVV Combined with KTN

**Figure 3:**
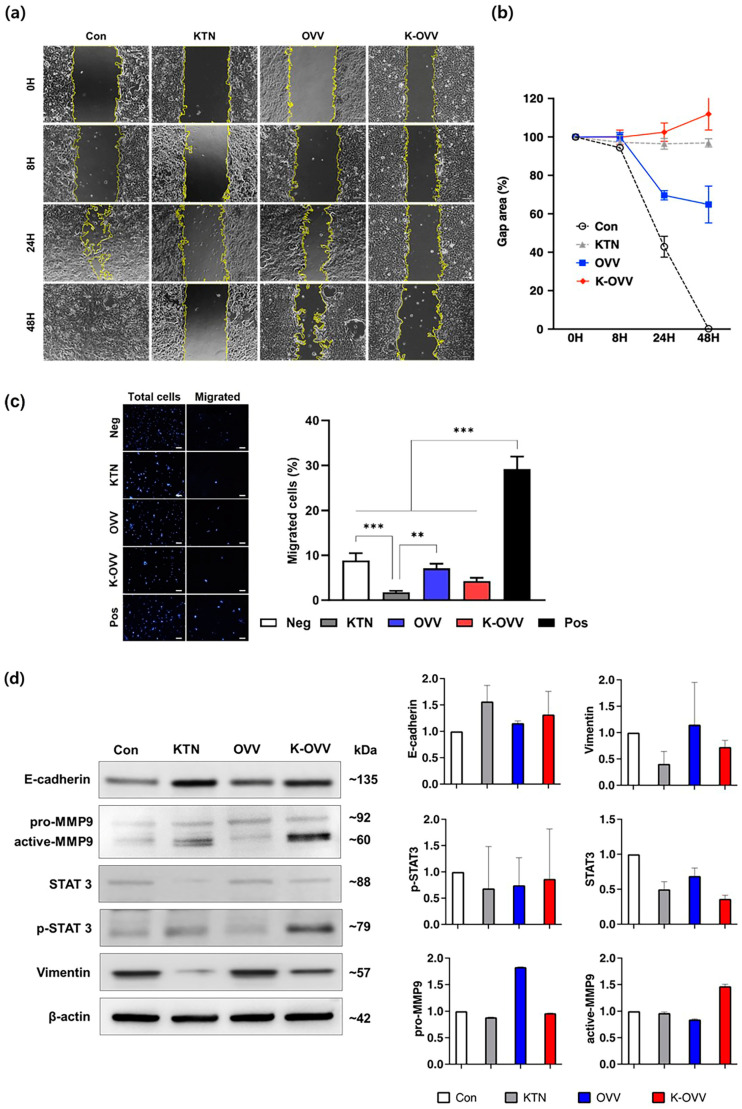
The in vitro anti-migratory effect of the OVV combined with KTN. The scratch wound migration assay of 4T1 mouse breast cancer cells treated with KTN combined with the OVV (MOI 1). The cells were observed under a light microscope (**a**), and the migrated cells were quantified (**b**). The transwell cell migration assay of the 4T1 cancer cells treated with KTN combined with the OVV (MOI 1). The cells were observed under a fluorescence microscope and the migrated cells were quantified (**c**). The protein expression pattern in each group from the migration assay (**d**). Scale bars: 100 µm. The values are presented as the mean ± SD. A two-way ANOVA was used for the analysis of the migration assay data, and the Mann-Whitney U test was performed on the Western blot results. ** *p* < 0.01, and *** *p* < 0.001.

To assess the metastatic potential of the 4T1 cells following treatment with the OVV combined with KTN, the migratory ability of the cells was evaluated by monitoring the relative scratch gap observed via images at various time points (Figure 3a). The DPBS-treated control group exhibited the fastest cell migration over time, effectively closing the scratch gap within 48 h. Conversely, the K-OVV group demonstrated the lowest cell migration in the cell-free gap until 24 h, with an inward recession area appearing after 48 h, further widening the gap (Figure 3b). Circular gap areas resembling the infectious centers were observed in the OVV group at 48 h, indicating virus-induced cell death in adjacent cells. This effect was more pronounced in the K-OVV group, where cell death was significantly greater. Only the K-OVV group exhibited an expanded cell-free gap area between 24 and 48 h, while other groups showed decreased gap areas. Similar trends were observed in the transwell cell migration assay (Figure 3c). Despite inducing migration with 10% FBS in the lower chamber, migration was similar to or lower than that of the negative control in all three treatment groups, where FBS was not added to the upper and lower chambers. The degree of migration inhibition followed the order of the KTN, K-OVV, and OVV group, confirming that migration was most inhibited when the KTN was administered.

To elucidate the anti-migratory mechanism of the formulation, the expression of the cell migration- and metastasis-related protein was evaluated (Figure 3d). The K-OVV group and the KTN group exhibited similar expression patterns, which were distinct from those observed in the OVV group. Specifically, epithelial cadherin (E-cadherin), an adhesion molecule involved in tether formation and cluster coherence maintenance, was significantly upregulated in K-OVV-treated 4T1 cells compared to the control and OVV groups [28]. Vimentin, a filament protein associated with invasion and metastatic potential, was significantly downregulated in the K-OVV group compared to the control group. Matrix metalloproteinase 9 (MMP9), responsible for extracellular matrix degradation facilitating tumor invasion and metastasis, was also downregulated in the K-OVV group. Additionally, STAT3, which plays a critical role in cancer progression by regulating the genes involved in cell growth, survival, and metastasis, was significantly suppressed in the K-OVV group compared to the control and OVV groups. STAT3 promotes EMT, a key factor in cancer metastasis, by reducing E-cadherin and increasing vimentin and MMP9 expression [29,30]. The suppression of STAT3 in the K-OVV group is associated with the increased expression of E-cadherin and the decreased expression of vimentin and MMP9. This suggests, that KTN enhances the antitumor efficacy of OVV by regulating the STAT3 signaling pathway. By inhibiting STAT3 signaling, the metastatic potential is reduced, and cell adhesion properties are increased, ultimately inhibiting cancer cell migration and invasion. These findings demonstrate that STAT3 plays a crucial role in regulating genes involved in cancer cell growth and survival, and that KTN can synergize with OVV to produce more effective anticancer outcomes. These in vitro results suggest that KTN suppresses 4T1 cell metastasis through the downregulation of STAT3 signaling. By inhibiting STAT3, KTN not only reduces the expression of genes that promote metastasis but also enhances the stability and retention of oncolytic viruses within the tumor microenvironment, thereby improving overall therapeutic outcomes.

### 2.4. In Vivo Tumor-Suppressing Effect of OVV Combined with KTN

**Figure 4:**
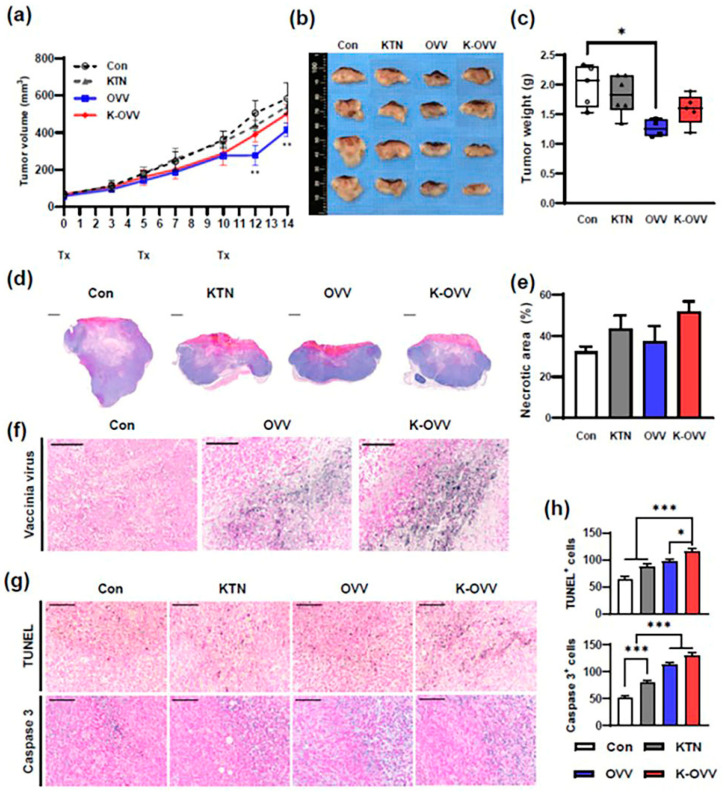
The in vivo tumor-suppressing effect of the OVV combined with KTN. A tumor growth curve (**a**), representative images of tumor samples (**b**), and tumor weight (**c**) of the 4T1 tumors treated with DPBS (Con), the oncolytic vaccinia virus (OVV), a 1% keratin solution (KTN), and the OVV with KTN (K-OVV). The representative images of the tumor sections stained with H&E (**d**) and measurements of the tumor necrotic lesions (**e**). The representative images of IHC staining against vaccinia virus in tumor sections (**f**). The representative images of the TUNEL assay and the IHC staining against caspase 3 (**g**). The number of TUNEL-positive cells and caspase 3-positive cells (**h**). Scale bars: 2 mm in (**d**), 200 µm in (**f**), and 100 µm in (**g**). The values are presented as the mean ± SEM. A two-way ANOVA was used for the comparison of tumor growth and a one-way ANOVA was used for the comparison of tumor weight, necrotic area, TUNEL staining, and caspase 3 staining, which was followed by the Bonferroni post hoc test. * *p* < 0.05, ** *p* < 0.01, and *** *p* < 0.001.

To confirm the effect of the OVV combined with KTN on TNBC in vivo, a syngeneic 4T1 mouse model was used. Mice were intratumorally administered with DPBS (Con), a 1% (*w*/*v*) keratin solution in DPBS (KTN), 1 × 10^7^ pfu of the OVV in DPBS (OVV), and 1 × 10^7^ pfu of the OVV combined with a KTN solution (K-OVV). Tumor growth was notably slower in the OVV group and the K-OVV group compared to the Con and KTN groups until day 10. Following the third administration on day 10, tumor growth in the OVV group was transiently suppressed for two days. Tumor proliferation was significantly attenuated in the K-OVV group and OVV group on day 12 compared to that of the control group (*p* < 0.05 and *p* < 0.001, respectively), highlighting the tumor-suppressive effects of the OVV (Figure 4a). The tumor weights measured upon sacrifice on day 19 mirrored the tumor growth curve, with the lowest weights observed in the OVV group, followed by the K-OVV group (Figure 4b,c). Histological analysis revealed the formation of necrotic lesions in the central region of the tumors, with infiltrating growth into adjacent tissues (Figure 4d). The necrotic area was pronounced in the K-OVV group, followed by the KTN and OVV groups, compared to the Con group (Figure 4e).

To determine the virus-stabilizing effect of KTN, tumor tissues were stained with an anti-vaccinia virus monoclonal antibody. The results revealed the persistence of the virus even nine days after the last treatment, especially within the marginal zone of necrotic lesions (Figure 4f). The stronger staining in the K-OVV group indicated a higher retention of the virus following treatment with the OVV combined with KTN. Tumor cell apoptosis was enhanced in the K-OVV group, with widespread apoptotic staining observed throughout the tumor compared to limited staining near and within the necrotic area in the Con group (Figure 4g). The number of apoptotic tumor cells was also higher in the K-OVV group (Figure 4h).

**Figure 5:**
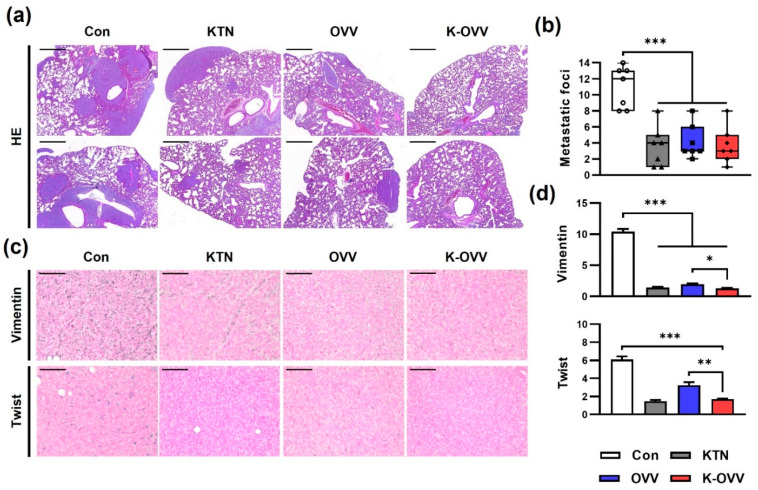
The in vivo anti-metastatic effect of the OVV combined with KTN. The representative images of metastatic lesions in the lung (**a**) and the number of metastatic foci per lung section (**b**). The representative images of the IHC staining against Twist and vimentin in tumor tissues (**c**) and the relative optical density of Twist positivity and vimentin positivity (**d**). Scale bars: 500 µm in (**a**) and 100 µm in (**c**). The values are presented as the mean ± SEM. A one-way ANOVA was followed by the Bonferroni post hoc test. * *p* < 0.05, ** *p* < 0.01, and *** *p* < 0.001.

Metastasis was exclusively observed in the lungs in all groups, and the degree of metastasis was quantified (Figure 5a). The number of metastatic foci was significantly lower in the three treatment groups compared to the control group (Figure 5b). Vimentin expression, indicative of the epithelial–mesenchymal transition (EMT), was significantly decreased in all of the treatment groups compared to the control group (Figure 5c,d). Twist, which is another EMT marker, was also decreased in all of the treatment groups, with the difference between the OVV and K-OVV groups becoming more pronounced. These results suggested that both the OVV and KTN suppressed the metastatic potential of 4T1 tumors by inhibiting EMT, with the effect being further enhanced by administration.

### 2.5. In Vivo Immunomodulatory and Antiangiogenic Effect of OVV Combined with KTN

**Figure 6:**
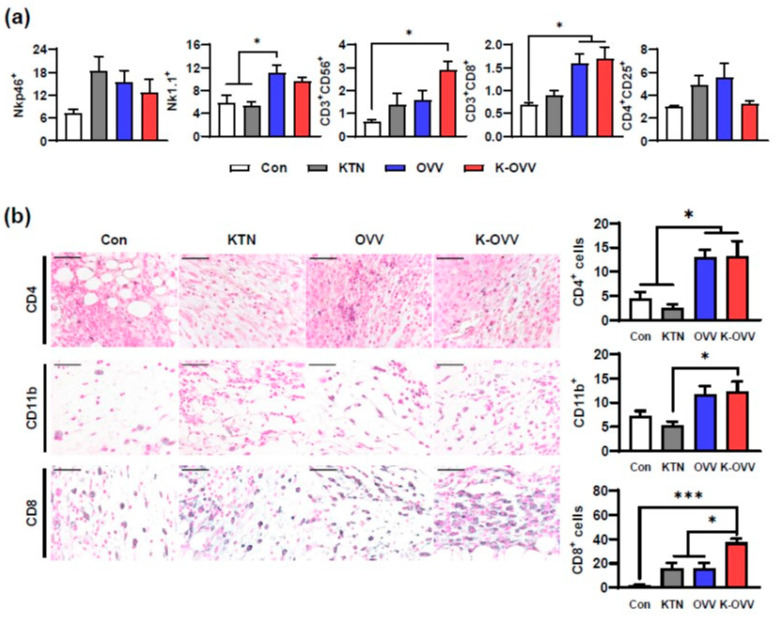
The in vivo immunomodulatory effect of the OVV combined with KTN. The flow cytometric analysis of peripheral blood (**a**). The representative images of the IHC staining of tumor sections against CD4, CD8, and CD11b as well as the number of CD4-, CD8-, and CD11b-positive cells, respectively (**b**). Scale bars: 50 µm. The values are presented as the mean ± SEM. A one-way ANOVA was followed by the Bonferroni post hoc test. * *p* < 0.05 and *** *p* < 0.001.

In addition to tumor-specific cell lysis, the OVV is known to exert its antitumor effects through immune modulation and the inhibition of angiogenesis [10]. To investigate whether these effects were influenced by concomitant administration with KTN and to evaluate systemic changes in the immune system, peripheral blood mononuclear cells were analyzed by flow cytometry. The OVV group exhibited an increase in Nk1.1+ natural killer cells compared to the Con and KTN groups (Figure 6a). Moreover, the K-OVV group showed a significant increase in CD3+ CD56+ natural killer T cells compared to the Con group, suggesting an additive effect of the OVV and the KTN. CD3+CD8+ cytotoxic T cells were also increased in both the OVV and K-OVV groups, indicating that the OVV induced a systemic increase in NK cells, NKT cells, and cytotoxic T cells, and that the KTN did not interfere with these immunomodulatory effects of the OVV.

To confirm the systemic tumor-suppressive effects, the immune response was evaluated by assessing tumor-infiltrating leukocytes. The infiltration of CD4+ regulatory T cells was increased in both the OVV group and the K-OVV group (Figure 6b). Additionally, CD11b+ monocyte lineage cells showed a slight increase in the K-OVV group. However, the increased infiltration of CD8+ cells in the surrounding stroma was more prominent than that of CD4+ cells. Notably, the K-OVV group exhibited a marked increase in CD8+ immune cell infiltration, indicating an additive effect of the OVV and KTN. While the OVV alone enhanced T cell responses, the administration of the K-OVV resulted in a more favorable immune profile with a greater increase in CD8+ cytotoxic T cells. These findings demonstrated that KTN combined with the OVV enhanced both local and systemic immune responses, recruiting effector T cells that suppress tumor growth.

**Figure 7:**
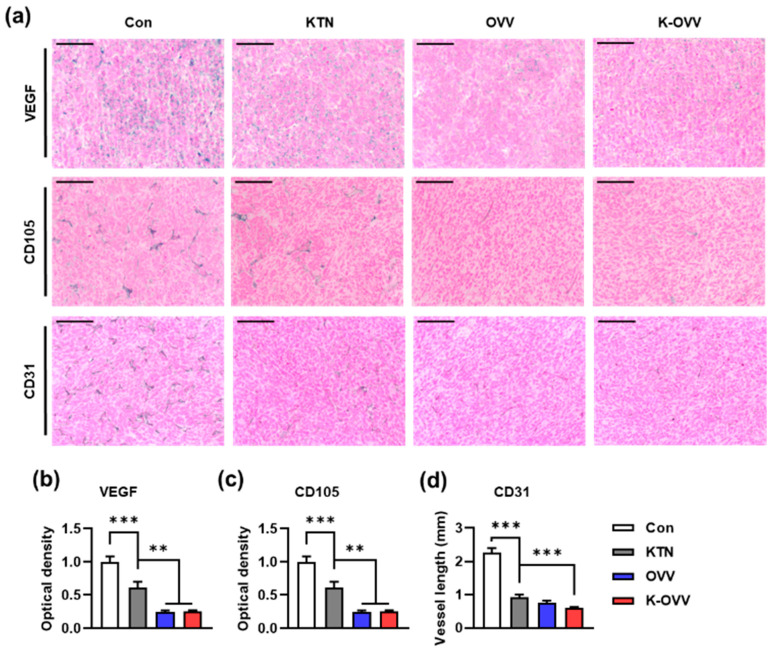
The in vivo antiangiogenic effect of the OVV combined with KTN. The representative images of the IHC staining for the VEGF, CD105, and CD31 (**a**). The relative optical density of VEGF (**b**) and CD105 positivity (**c**), and the CD31 positive vessel length per HPF (magnification: 200×) in tumor tissue (**d**). Scale bars: 100 µm. The values are presented as mean ± SEM. ** *p* < 0.01 and *** *p* < 0.001 as per one-way ANOVA followed by the Bonferroni post hoc test.

To evaluate tumor vascularity, the expression of angiogenic factors in tumor cells was assessed, and vessel length in the tumor tissue was examined. VEGF, a major factor in neo-vessel formation, was highly expressed throughout tumor cells in the control group (Figure 7a). KTN treatment alone suppressed VEGF expression, and further suppression was observed in both the OVV and K-OVV groups (Figure 7b). The staining of the neo-vessel marker CD105 and mature vessel marker CD31 showed a similar pattern as seen in the VEGF. Therefore, these results suggest that KTN alone suppressed vascular formation, and concomitant administration with the OVV did not interfere with its antiangiogenic effect.

## 3. Discussion

Triple-negative breast cancer (TNBC) poses a significant challenge in oncology due to its aggressive behavior, propensity for early metastasis, and high recurrence rates [3]. Traditional chemotherapy often proves insufficient in effectively treating TNBC, highlighting the urgent need for innovative therapeutic approaches [31]. Among these, oncolytic viruses have emerged as promising candidates for combating metastatic and chemo-resistant tumors. Their advantage lies in their ability to target both primary tumors and metastatic lesions while augmenting the antitumor immune response to prevent recurrence. However, optimizing oncolytic virotherapy presents several hurdles, including evading host immunity to the vaccinia virus, enhancing systemic delivery, and improving intratumoral spread [7]. To tackle these challenges, we investigated a strategy involving the utilization of keratin in conjunction with oncolytic virotherapy.

Previous research has explored keratin-based delivery systems for various therapeutic agents in cancer treatment, such as doxorubicin and photosensitizers for photodynamic therapy [21,32,33]. Additionally, keratin has been shown to promote intracellular nitric oxide production, sensitizing tumor cells and enhancing the anticancer efficacy of chemotherapeutic drugs [21,32,34]. However, prior studies primarily focused on evaluating the drug delivery capacity of keratin without probing its intrinsic anticancer properties. In contrast, our study diverges from this approach by employing keratin as a delivery system that enhances the stability of oncolytic viruses. Unlike previous studies that treated keratin as a negative or vehicle control, our findings demonstrate that KTN, when administered alone, exerts antitumor effects by suppressing tumor growth and metastasis. These results align with other investigations into the anticancer effects of hair proteins on various cancer cell lines, including melanoma, lymphoma, and bladder cancer cell lines [22]. Nevertheless, further elucidation is needed regarding the underlying mechanism of KTNs anticancer activity.

Oncolytic viruses such as the vaccinia virus exert their antitumor effects through multiple mechanisms, including modulation of the host immune response, direct viral oncolysis, and induction of vascular collapse [35]. In our study, KTN and OVV exhibited a clear synergistic effect, outperforming either agent alone. This synergy is significant, as KTN not only stabilizes OVV within the tumor microenvironment but also modulates key pathways like STAT3, dnhancing OVV’s ability to suppress tumor growth and metastasis. These findings build on previous studies that focused solely on OVV or KTN, demonstrating the added benefit of combining these therapies. While our results highlight the therapeutic potential of combining KTN with OVV, further studies are needed to evaluate the long-term stability of KTN-OVV formulations and their efficacy in other cancer types. Additionally, combining KTN-OVV therapy with other immunotherapies could further improve outcomes.

Epithelial–mesenchymal transition (EMT) is a crucial process implicated in cancer metastasis, including breast cancer [36,37]. Our study demonstrated that KTN inhibits EMT by suppressing STAT3 signaling, which is consistent with previous findings in pancreatic cancer [38]. STAT3 is known to regulate genes involved in cell growth, survival, and metastasis. By modulating STAT3 activity, KTN enhances the antitumor efficacy of the OVV through better immune modulation and direct tumor cell targeting [29,30]. This implies that the KTNs EMT-suppressing effect may extend beyond TNBC, offering potential therapeutic implications in other cancer types characterized by EMT and metastasis. Further exploration of KTNs mechanisms in regulating EMT may unveil new avenues for therapeutic interventions targeting metastasis across a broader spectrum of cancers.

While our study utilized the modified vaccinia virus JX-929, which carries a cytosine deaminase gene enabling the conversion of 5-fluorocytosine to 5-fluorouracil, the potential synergistic effects of KTN and 5-fluorocytosine combination therapy were not evaluated. Future investigations should explore the synergistic potential of KTN in combination with 5-fluorocytosine to optimize therapeutic outcomes.

Previous clinical studies have demonstrated that intratumoral injection of vvDD, a double-deleted vaccinia virus, induces systemic immune responses characterized by elevated CD4+ and CD8+ T cells [39,40]. Our findings align with these observations, as treatment with the OVV alone resulted in a systemic increase in various immune cell populations, including NK cells, NKT cells, and cytotoxic T cells. Remarkably, combining KTN with the OVV (K-OVV) further augmented the immune response by recruiting a greater number of effector T cells, particularly CD8+ cytotoxic T cells. This suggests that KTN enhances virotherapy efficacy not only by stabilizing the virus but also through its intrinsic antitumor effects, thereby enhancing overall treatment efficacy.

The OVV can directly infect tumor-associated endothelial cells, leading to tumor vasculature destruction in addition to targeting tumor cells [15,16,17]. Consistent with previous research, our results demonstrate that the OVV exerts an antiangiogenic effect by targeting the VEGF, a key regulator of neo-vessel formation. The combination of KTN and the OVV resulted in a significant reduction in VEGF expression within the tumor, leading to decreased tumor vascularization and neoangiogenesis. Furthermore, KTN alone also exhibited an antiangiogenic effect, albeit to a lesser extent than the OVV. These findings suggest that KTN-OVV therapy could provide a novel approach for treating aggressive cancers like TNBC, potentially reducing the need for more toxic chemotherapy regimens. Future clinical studies could explore its use in combination with existing therapies to improve patient outcomes. This additional antiangiogenic property of KTN, possibly mediated through STAT3 signaling suppression, positions it as a potential candidate for combination therapy with other anticancer agents lacking antiangiogenic properties [41]. While our study utilized the modified vaccinia virus JX-929, further research should evaluate the long-term efficacy and stability of KTN-OVV formulations in a broader range of cancer types. Additionally, exploring the combination of KTN-OVV therapy with other immunotherapies may further enhance therapeutic outcomes.

## 4. Materials and Methods

### 4.1. Materials

Human hair samples were obtained from healthy donors with non-chemically modified natural black-colored hair. Chemicals including urea, thiourea, 2-mercaptoethanol, Trizma base, hydrochloric acid (HCl), and an Annexin V staining kit were purchased from Sigma-Aldrich (St. Louis, MO, USA). Peracetic acid was purchased from Dongmyung ONC (Busan, Republic of Korea). The vacuum-driven filtration system was purchased from Merk Millipore. Pretreated RCtubing (MWCO: 12–14 kDa) was purchased from Spectrum Laboratories, Inc. (Rancho Dominguez, CA, USA). The genetically engineered OVV (JX-929) was kindly provided by SillaJen Inc. (Busan, Republic of Korea). A QIAamp MinElute virus spin kit was purchased from Qiagen (Venlo, The Netherlands). Cell culture reagents, including RPMI-1640, trypsin-EDTA, fetal bovine serum (FBS), penicillin–streptomycin (PS), and Dulbecco’s phosphate-buffered saline (DPBS), were purchased from GIBCO BRL (Invitrogen Co., Waltham, MA, USA). The cell counting kit-8 was purchased from Dojindo Molecular Technologies, Inc. (Rockville, MD, USA).

### 4.2. Extraction of KTN from Human Hair

Keratin (KTN) was extracted from human hair via the Shindai method, as previously described [23,24]. Briefly, 20 g of 15-cm-long human hair samples were washed with light detergent and rinsed with distilled water several times. Delipidization was performed by soaking the samples in a solution containing chloroform and methanol (2:1, *v*/*v*) for 24 h, followed by washing with distilled water until all of the remains were removed. The hair samples were air-dried and then soaked in a 2% peracetic acid solution for 12 h at 37 °C. The KTN extraction was carried out by placing the hair samples in a Shindai solution containing 5% mercaptoethanol, 5 M urea, 2.6 M thiourea, and 25 mM Trizma base (pH 8.5). The supernatant of the KTN solution was dialyzed using a cellulose membrane (MWCO: 12–14 kDa) against distilled water, and the resulting KTN was lyophilized and stored at −80 °C.

### 4.3. Characterization of KTN

The molecular weight of the extracted KTN was measured using sodium dodecyl sulfate–polyacrylamide gel electrophoresis (SDS-PAGE). The KTN samples (1% and 2% *w*/*v* in DPBS) were reduced using the 2× SDS-PAGE loading buffer (Biosesang, Seongnam, Republic of Korea) by heating at 95 °C for 5 min, and were then subjected to electrophoresis on a 5% stacking gel and a 12% running Bio-Tris gel at 200 V for approximately 30 min. The proteins in the gel were stained with Coomassie Brilliant Blue. The surface morphology of the KTN was examined using a scanning electron microscope (S-4700; Hitachi, Tokyo, Japan) after preconditioning the samples using a Pt sputter-coating system (IB3; Eiko, Tokyo, Japan).

### 4.4. Stability of OVV in KTN Solution

To assess the stability of the OVV in the KTN solution, the OVV (1 × 10^7^ pfu) was mixed into a 1% KTN solution, or DPBS, and incubated in a 24-well plate under standard cell culture conditions (37 °C, 5% CO_2_). Viral DNA was extracted using QIAamp MinElute Virus Spin Kit (Qiagen), and real-time qPCR analysis was performed using SYBR Green (Invitrogen) on an ABI Prism 7500 (Applied Biosystems, Waltham, MA, USA) to quantify the viral DNA. Primers targeting the E9L region of the vaccinia virus were used for the qPCR analysis.

### 4.5. Release of OVV in KTN Solution

For the release of the OVV in the KTN solution, the OVV (2 × 10^3^ pfu) was mixed into a 1% KTN solution, or DPBS, and placed on a 3 μm pore size hanging insert. After shaking incubation for 30 min, the hanging insert-loaded plates were incubated in standard culture conditions. The released virus was collected from the PBS underneath the hanging insert at specified time points (0, 0.5, 1, 2, 3, 6 24, 48, and 72 h) after shaking, and the quantification was performed as described in Section 2.4.

### 4.6. In Vitro Tumor Cell Viability Test

The viability of the 4T1 (mouse breast cancer cell line) cells was assessed using the cell counting kit (CCK)-8 assay. The cells were seeded into the wells of a 96-well plate and treated with various concentrations of KTN or KTN combined with the OVV. The viability was determined at different time points using the CCK-8 assay, according to the manufacturer’s instructions.

### 4.7. In Vitro Tumor Cell Imaging of KTN-Combined OVV

To assess the anti-carcinogenic activity of the KTN-entrapped OVV, the mouse breast cancer cell line 4T1 was seeded into a 96-well plate at a density of 2 × 10^3^ cells/100 μL. After 24 h of incubation, the cells were treated with 20 μL of DPBS, 1% KTN, the OVV at MOI = 1, and 1% KTN containing the OVV at MOI = 1. Following the addition of 180 μL of medium to each well, cells were further incubated for 48 h. Subsequently, cell death was assessed using the Annexin V-FITC Apoptosis Detection kit (Sigma-Aldrich). The nuclei were stained with 4′, 6-diamidino-2-phenylindole (DAPI) for 3 min and observed under a fluorescence microscope with the appropriate light source.

### 4.8. In Vitro Tumor Cell Migration of KTN-Combined OVV

Cell migration was assessed through scratch wound healing assay and transwell cell migration assay. For the scratch wound healing assay, 4T1 cells were cultured in 6-well plates at a seeding density of 6 × 10^5^ cells/mL/well. Upon achieving confluent monolayers, a scratch wound was created using a commercial scratcher (SPL, Pocheon, Republic of Korea) to generate a gap between monolayers. Subsequently, 200 μL of DPBS, 1% KTN, the OVV at MOI = 1, or 1% KTN containing the OVV at MOI = 1 were added to the wells. After incubation for 8, 24, and 48 h, the gap area between cell monolayers was imaged at 40× magnification under a light microscope, and the area was quantified using ImageJ software (Version 1.51).

For the transwell cell migration assay, 2 × 10^4^ cells were cultured on the commercial transwell membranes with an 8 μm pore size and 0.33 cm^2^ of growth area (SPL). In the Negative control (Neg) group, FBS was not added to either the upper and lower chambers, while in the KTN, OVV, K-OVV, and Positive control (Pos) groups, FBS was not added to the upper chamber and 10% FBS was added only to the lower chamber. An amount of 20 μL of DPBS, 1% KTN, the OVV at MOI = 1, or 1% KTN containing the OVV at MOI = 1 was added to the upper chamber of the wells. After 24 h of incubation, the total and migrated cells were stained with DAPI and counted using ImageJ software.

### 4.9. Western Blot Analysis

Total protein from the 4T1 cells used in the migration assay was extracted using an M-PER (Thermo Fisher Scientific, Waltham, MA, USA) or RIPA (Cell Signaling Technology, Danvers, MA, USA) extraction buffer with a protease inhibitor cocktail (Thermo Fisher Scientific). The protein concentration was determined using a protein assay kit (Bio-Rad, Hercules, CA, USA). An amount of 40 μg of total protein was loaded onto Tris-glycine gels and transferred to polyvinylidene fluoride membranes. The membranes were incubated with primary antibodies against E-cadherin, phosphor-STAT3, STAT3, β-actin (Cell Signaling Technology), anti-MMP9 antibody (Abcam, Cambridge, UK), and anti-vimentin (Dako). Subsequently, membranes were incubated with horseradish peroxidase-conjugated secondary antibodies (Cell Signaling Technology) and immunoreactive bands were visualized using a CCD camera (Fusion solo 6S.WL; Vilber, Marne-la-Vallée, France) with a chemiluminescent substrate (Clarity Western ECL Substrate; Bio-Rad). Band quantification was performed using ImageJ software with β-actin used for standardizing the protein content per loaded sample.

### 4.10. Animal Experiments

The experimental procedures were conducted following the protocols approved by the Institutional Animal Care and Use Committee of Konkuk University (KU17066). To establish the syngeneic 4T1 model, 5 × 10^5^ 4T1 tumor cells mixed with Matrigel (1:1, *v*/*v*) were injected into the fourth mammary fat pad of female 6-week-old BALB/c mice. The tumor sizes were monitored using calipers, and treatment initiation occurred when the tumor size reached 60–80 mm^3^. Mice were randomly divided into four groups (*n* = 6 per group) including a Control (Con) group, which received a DPBS (50 μL) injection, a virus group, which received an OVV (1 × 10^7^ pfu/50 μL) injection, a KTN group, which received a keratin solution (1% *w*/*v* in DPBS, 50 μL) injection, and a KTN+V group, which received an OVV (1 × 10^7^ pfu/50 μL)-loaded KTN solution injection. All treatments were administered intratumorally. It is noted that the OVV has shown effectiveness with single or multiple administrations at 1 × 10^7^ to 1 × 10^8^ pfu or more, sometimes lower for armed viruses [42,43,44]. To determine the viral delivery using KTN without aggressive virotherapy, a relatively lower dose of 1 × 10^7^ pfu was injected. Mice were euthanized at 19 days post-treatment, and tumor tissues along, with vital organs, were collected for histological analysis. Peripheral blood was also collected for flow cytometric analysis.

### 4.11. Flow Cytometric Analysis

Peripheral blood samples were collected, and red blood cells were lysed with RBC lysis buffer. Cells were washed in PBS containing 1% FBS and then stained with monoclonal mouse anti-CD8 (Santa Cruz Biotechnology, Dallas, TX, USA), rabbit anti-CD4 (Santa Cruz Biotechnology), rabbit anti-CD3 (Abcam), mouse anti-CD25 (Thermo Fisher Scientific, Waltham, MA, USA), rat anti-Gr1 (Thermo Fisher Scientific), rabbit anti-CD11b (Santa Cruz Biotechnology), rat anti-Nkp46 (BioLegend, San Diego, CA, USA), mouse anti-Nk1.1 (Thermo Fisher Scientific), and mouse anti-CD56 (Abcam) antibodies. After fixation with 4% paraformaldehyde, cells were incubated with FITC or Alexa Fluor-conjugated antibodies (Santa Cruz Biotechnology). Flow cytometric analysis was performed using FACSCalibur (BD Biosciences, Franklin Lakes, NJ, USA), analyzing 10,000 cells per sample.

### 4.12. Histological Evaluation and Immunohistochemistry

Tumor tissues and vital organs, including lung, heart, liver, kidney, and spleen, were harvested and fixed with 10% neutral buffed formalin (BBC Biochemical, McKinney, TX, USA). Sections (4 μm in thickness) were prepared and stained with hematoxylin and eosin (H&E) for histological examination. For immunohistochemistry, sections were stained using anti-CD11b (FineTest, Wuhan, China), anti-CD8 (Santa Cruz Biotechnology), anti-CD4 (Santa Cruz Biotechnology), anti-vaccinia virus (Abcam), anti-caspase 3 (Abcam), anti-VEGF (Abcam), anti-CD31 (Abcam), anti-CD105 (Abcam), anti-Twist (Abcam), and anti-vimentin (Abcam) antibodies. Staining was visualized using a Vectastain^®^ Elite ABC-Peroxidase kit (Vector Laboratories, Burlingame, CA, USA), and the sections were counterstained with Nuclear Fast solutions (Vector Laboratories). Apoptotic cells were detected using terminal deoxynucleotidyl transferase-mediated dUTP nick-end labeling (TUNEL; Roche Diagnostics, Basel, Switzerland) according to the manufacturer’s instructions, followed by visualization using Vector SG and counterstaining with Nuclear Fast solutions. The quantification of positive cells, mean optical density, and vessel length was conducted in multiple fields using ImageJ.

### 4.13. Statistical Analysis

Statistical analyses were performed using GraphPad Prism software (version 10.3.1). Data are presented as mean ± standard deviation (SD) or standard error of the mean (SEM), as indicated. Statistical significance was determined using two-way ANOVA followed by Bonferroni post-hoc test, one-way ANOVA with Bonferroni post-hoc test, or the Mann-Whitney U test. A *p*-value < 0.05 was considered statistically significant. All experiments were conducted at least three times independently to ensure reproducibility and reliability of the results.

## 5. Conclusions

In summary, our study underscores the potential of KTN as a valuable adjunct for enhancing the antitumor efficacy of the OVV. In vitro experiments demonstrated that KTN enhances OVV stability without compromising its distribution. Both KTN and the OVV exerted antitumor effects, inducing apoptosis and necrosis. Their combined administration showed generally additive effects. Moreover, KTN inhibited cancer cell migration by suppressing metastasis-related protein expression and EMT via STAT3 downregulation. In vivo experiments using a syngeneic TNBC mouse model validated the oncolytic and anti-metastatic effects of KTN and the OVV. Combining KTN with the OVV improved OVV distribution within tumors, resulting in increased tumor cell infection and killing. Additionally, KTN enhanced the OVVs suppressive effect on EMT and elicited a robust immune response mediated by cytotoxic T cells against tumors. Overall, our findings suggest that KTN can enhance OVV efficacy by improving distribution, suppressing EMT, and modulating the immune response, without interfering with the OVVs major antitumor mechanisms. These findings underscore the potential of KTN as a delivery platform for oncolytic virotherapy and advocate for its exploration in combination with other immunotherapeutic modalities.

## Data Availability

The original contributions presented in the study are included in the article, further inquiries can be directed to the corresponding author.
